# The Latest Practices in Culture‐Free Detection of Bacteria in Water, From Sampling to Membrane Filtration and DNA Extraction: A Systematic Review

**DOI:** 10.1002/mbo3.70119

**Published:** 2025-11-04

**Authors:** Radu Ovidiu Togănel, Cristina Nicoleta Ciurea, Anca Cighir, Anca Delia Mare, Razvan Lucian Coșeriu, Camelia Vintilă, Dragoș Constantin Cucoranu, Adrian Man

**Affiliations:** ^1^ Microbiology Department George Emil Palade University of Medicine, Pharmacy, Science, and Technology of Targu Mures Tărgu Mureș Romania; ^2^ Doctoral School of Medicine and Pharmacy George Emil Palade University of Medicine, Pharmacy, Science and Technology of Târgu Mureș Târgu Mure Romania; ^3^ Independent researcher Târgu Mures Romania

**Keywords:** bacteria, DNA extraction, filtration, sampling, water

## Abstract

The advancement of molecular biology in water research, combined with the lack of standardization in this area of research, exposed the need for presenting the different methodological approaches available to researchers. The aim of the article is to identify and critically discuss the water filtration methods for culture‐free bacterial DNA extraction. A systematic review was conducted on PubMed and Web of Science, according to the 3Cochrane Handbook recommendations and PRISMA 2020 Checklist. The initial search retrieved 513 articles, and 53 were included with a multi‐step approach screening (title, abstract, full text). Outcomes of interest involved details about sampling, filtration methods and DNA extraction. The most reported sampling methods were using containers, especially of 1000 mL. Filtration was performed using membrane filters, the majority of the studies using Polyethersulfone (PES) membranes with the pore size of 0.22 µm. Samples were prepared for extraction either with the help of an enzymatic pretreatment (protease, proteinase K, lysozyme, or lysostaphin), physical methods (bead‐beating, centrifugation, vortexing, sonication, heating, freeze‐thaw cycles), or detergent pretreatment. The preferred method for extraction was using commercially available kits, although some of the studies described in‐house protocols. The methods must be adapted to the scientific scope. The review summarizes the existing methods and critically appraises their utility and promotes advancement in the field of environmental molecular biology.

## Graphical abstract

Multiple methodologies are available for researchers for culture‐free bacterial DNA detection from water. Sampling can be performed by using containers, pumps or specialized devices. Filtration can done by using various membrane filters materials and pore sizes. Bacterial DNA extraction with commercial kits or in‐house protocols.

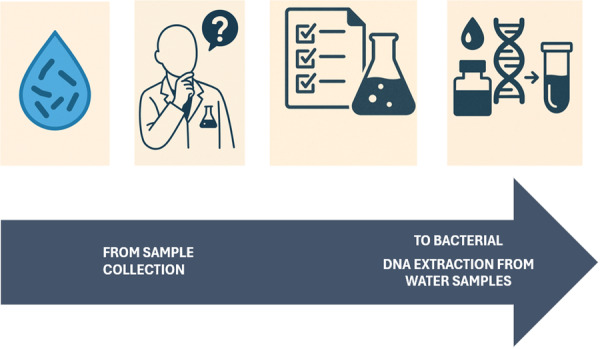

## 1 Introduction

Water is the source of life and historically all of the civilizations developed near water areas. Despite its indispensability, not everybody has access to safe drinking water, even in 2025. Bacteria polluting water are diverse: species of *Vibrio*, *Salmonella, Shigella, Escherichia coli*, enterococci and many other genera, compromising the health of millions every year (Cabral 2010). Bacteria might contaminate water sources, thus numerous infectious diseases are waterborne (Pandey et al. 2014). Studies assessing the bacterial contamination of water are more and more popular, as they are important from a public health perspective, but also for the surveillance of antimicrobial resistance. In the context of a changing climate, increased urbanization, and pressure on freshwater resources, understanding the microbial quality of water is critical. Protecting water sources from microbial contamination is relevant not only to preventing disease outbreaks, but also to ensuring long‐term environmental and population health.

The current literature on the topic of microbiological and molecular analysis of water (either groundwater, surface water, wastewater, or finished water) is rich and diverse, reflecting a broad spectrum of methodologies and findings, with various contributions. Lack of consensus regarding the best methodological approach for experimental studies aiming to extract bacterial DNA from water, without an a priori culturing step, highlights the need for providing up to date information on the topic. This lack of standardization poses significant barriers to data reproducibility, comparative analysis, and the implementation of molecular methods in routine water quality monitoring. Thus, identifying efficient, reliable, and scalable protocols for bacterial DNA extraction is relevant both to research laboratories and to public health institutions.

To the best of our knowledge, there are no other systematic reviews that consolidate the various methodologies for water analysis via filtration into a structured, step‐by‐step approach.

The aim of the study is to identify the current water filtration methods that can be used further for bacterial DNA extraction. The study provides ground‐based information that can be used for future molecular and epidemiological studies. By synthesizing the available techniques, this review contributes to the optimization of water microbiology protocols, facilitating more standardized decisions in the context of outbreak investigations, antimicrobial resistance surveillance, and environmental health.

## 2 Material and Method

A systematic review was carried out respecting the Cochrane Handbook recommendations and PRISMA 2020 Checklist.

Articles were eligible if they met the following inclusion criteria: English‐written peer‐reviewed original research articles, published between 2014 and 2024, reporting on water filtration methods, DNA extraction methods and/or DNA amplification methods, directly from the sample (not involving a culturing step).

Exclusion criteria: case reports, review articles, studies reporting outside of the aim of the review, studies not mentioning the filtration or the extraction method, or studies with insufficient data.

The search was conducted on PubMed and Web of Science using the query “(dna[Title/Abstract] AND extraction[Title/Abstract] AND bacteria[Title/Abstract] AND water[Title/Abstract])”.

Study selection followed a multi‐step approach: title screening, abstract screening and full‐text screening. Each step was documented on the PRISMA flow diagram. Every step of the selection was carried out manually and independently by two reviewers (R.O.T. and A.C.), while conflicts were resolved by a third reviewer (C.N.C).

From each included study, the following data were retrieved: sampling method, number of samples, quantity of each sample, type of water sampled, filtration method and volume, membrane type and pore size, whether the DNA extraction was performed directly from the filter or preceded by sample processing using a buffer/enzyme/physical method, and the extraction method used to obtain the DNA.

Data extraction was carried out by one investigator and checked out by a second one. The final database was obtained by consensus by at least two investigators. Data analysis was carried out using spreadsheet software (Microsoft® Excel).

## 3 Results

**Figure 1:**
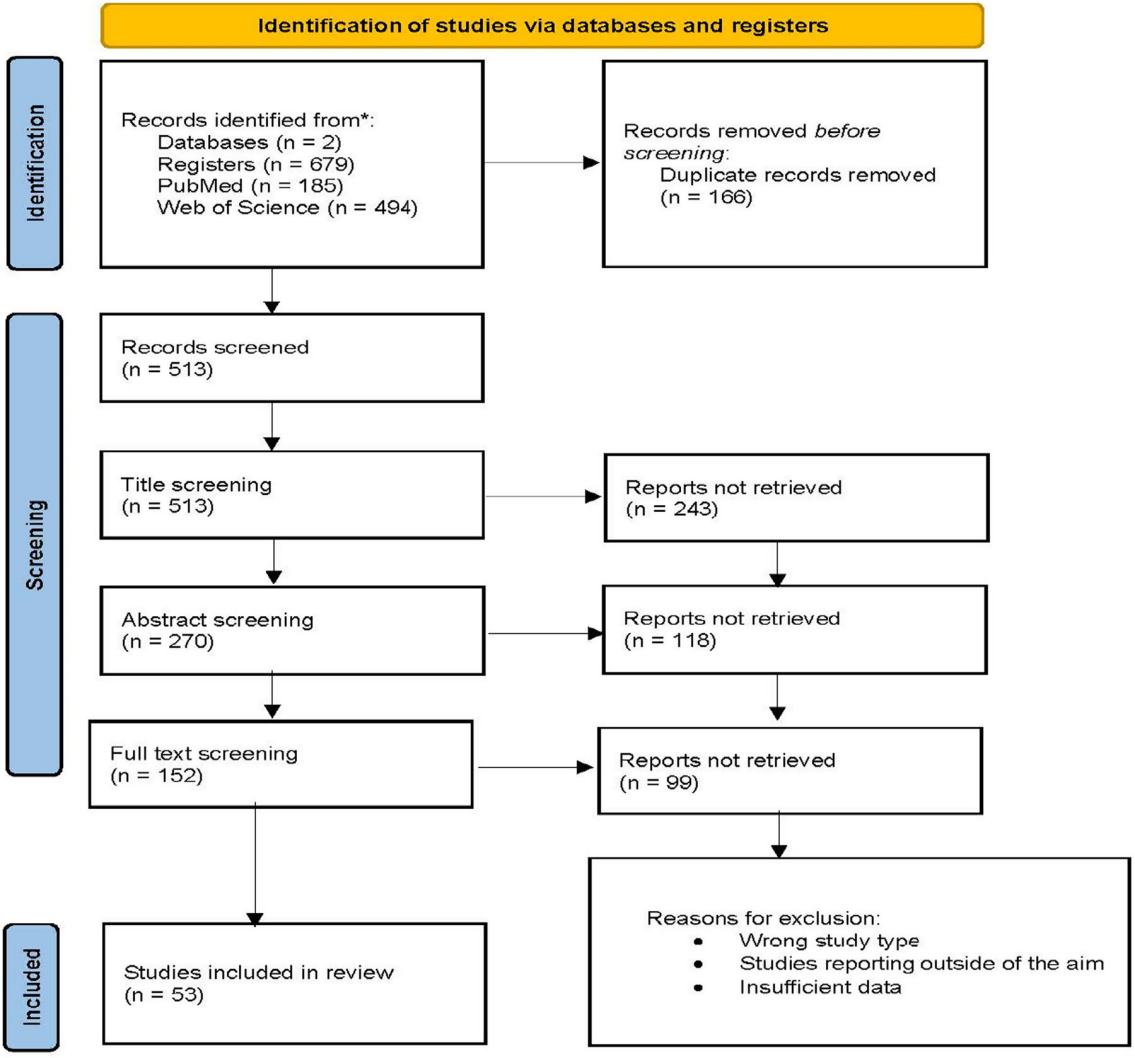
PRISMA 2020 flow diagram.

Study selection (PRISMA flow chart): Figure 1.

After the search and removal of the duplicates, 513 articles were identified and advanced into the title screening process. During the title screening, 270 articles were further directed to the abstract screening process. The full text screening included 152 articles, from which, 53 were finally included in the review. The entire process was documented in the Prisma Flow Chart (Figure 1).

### 3.1 Sampling Method

Most studies reported on water sampling methods used for further extraction of bacterial nucleic acids (47 out of 53; 88.67%). Water was collected directly from the water source mostly by using a container (*n* = 43; 81.13%), although some studies reported the possibility to use a pump (*n* = 2; 3.77%) or a horizontal water sampler (*n* = 1; 1.88%). In one study (Lee et al. 2019), the water used for filtration was artificially spiked with pre‐cultured bacteria.

Containers consisted of simple sterile recipients like plastic bottles (such as polypropylene) or glass bottles. Water was collected in containers in volumes ranging from 100 mL to 100 L (Table 1).

**Table 1:** The quantity in mL of the samples collected and the quantity filtered by membranes in mL.

Sample quantity (mL)	100–500		501–1000		1001–2000		2001–4000		4001–10,000		> 10,000	
	No of studies	Reference	No of studies	Reference	No of studies	Reference	No of studies	Reference	No of studies	Reference	No of studies	Reference
	10	(Lever et al. (2015); Shi et al. (2020); Mueller et al. (2014); Kambura et al. (2016); Cao et al. (2018); Deshmukh et al. (2019); Fisher et al. (2020); Brooks et al. (2015); Bian et al. (2023))	24	(Walden et al. (2017); Kurniawinata et al. (2022); Chopyk et al. (2020); Cavallaro et al. (2024); Mueller et al. (2014); Vierheilig et al. (2015); Stojan et al. (2023); Yasar et al. (2021); Hoorzook and Barnard (2022); Baron et al. (2015); Fisher et al. (2020); Huang et al. (2017); Fecteau (2022); Hinthong et al. (2024); Anderson and Thompson (2022); Brandt and Albertsen (2018); Sthapit et al. (2022); Amirhooshang et al. (2014); Fernández‐Baca (2021); Bae et al. (2019); Smalls et al. (2023); Marti et al. (2014); Mochware et al. (2024); Yao et al. (2024))	5	(Deepnarain et al. (2020); Uprety et al. (2023); Arsand et al. (2020); Bae et al. (2019); Uprety et al. (2024))	4	(Gensberger et al. (2014); Putri et al. (2021); Bae et al. (2019); Echeverry‐Gallego et al. (2024))	3	(Djurhuus (2017); Yasar et al. (2021); Yun et al. (2016))	4	(Demkina et al. (2023); Putri et al. (2021); Stojan et al. (2023); Zavarzina et al. (2022))
Quantity filtered (mL)	< 100		100–250		251–500		501–1000		> 1000			
	No of studies	Reference	No of studies	Reference	No of studies	Reference	No of studies	Reference	No of studies	Reference		
	6	(Shi et al. (2020); Mueller et al. (2014); Stojan et al. (2023); Fisher et al. (2020); Sthapit et al. (2022); Yang et al. (2020))	12	(Lee et al. (2019); Mueller et al. (2014); Vierheilig et al. (2015); Yasar et al. (2021); Cao et al. (2018); Hoorzook and Barnard (2022); Bian et al. (2023); Fecteau (2022); Fernández‐Baca (2021); Mochware et al. (2024); Abreu‐Silva et al. (2023); Wirajana et al. (2024))	7	(Cavallaro et al. (2024); Yasar et al. (2021); Fernández‐Baca (2021); Smalls et al. (2023); Mochware et al. (2024); Yao et al. (2024); Abreu‐Silva et al. (2023))	11	(Demkina et al. (2023); Djurhuus. (2017); Gensberger et al. (2014); Stojan et al. (2023); Yasar et al. (2021); Oueslati (2022); Hinthong et al. (2024); Anderson and Thompson (2022); Amirhooshang et al. (2014); Fernández‐Baca (2021); Abreu‐Silva et al. (2023))	3	(Bautista‐de los Santos et al. (2016); Yun et al. (2016); Abreu‐Silva et al. (2023))		

Pumps are devices used for collecting water underneath the surface at 1 meter (OSMO portable pump and filtration system) (Wijaya. 2023), or for collecting long‐term water: 24 h using a peristaltic pump at a speed of 75 rpm, to collect large quantities of water (13–17 L) (Bautista‐de los Santos et al. 2016). The studies in which water was extracted by using a pump sampled from 1500 mL (Wijaya. 2023), up to 13,000–17,000 mL (Bautista‐de los Santos et al. 2016).

The Wildco, Model 1960‐H65 (Yulee, FL) horizontal water sampler is a device that allows deeper sampling (3 m below surface) (Walden et al. 2017).

Pre‐spiking the water with pre‐cultured bacteria was chosen because the researchers needed a controlled number of bacterial cells to validate a new extraction method (Lee et al. 2019)

Sampling volume differed across the studies, with a minimum value of 1 mL and a maximum of 100,000 mL, with most studies (*n* = 20; 37.73%) sampling 1000 mL. The filtered volumes were also different, with the lowest value at 1 mL and the highest, at 15,000 mL, but most of them being below 1000 mL (*n* = 36, 67.92%).

### 3.2 Filtration

Different membrane types are available for researchers to use when they are looking for a filtration method: membrane filters used with a vacuum pump (MCE—mixed cellulose esters, PES—polyethersulfone, cellulose nitrate, cellulose acetate, polycarbonate, porous glass, glass fiber, nylon, vinyl, alumina), or syringe filters (glass fiber [Watanabe et al. 2020], PES [Lee et al. 2019]) (Table 2).

**Table 2:** Different membrane types used, classified by size.

Membrane pore size	0.2 µm −0.3 µm		0.4 µm −0.6 µm		0.7 µm −0.8 µm		1 µm −2 µm		> 2 µm	
Membrane type	No. of studies	Reference	No. of studies	Reference	No. of studies	Reference	No. of studies	Reference	No. of studies	Reference
MCE	1	(Putri et al. (2021))	2	(Shi et al. (2020); Huang et al. (2017))	0		1	(Arsand et al. (2020))	0	
PES	12	(Lee et al. (2019); Wijaya. (2023); Bautista‐de los Santos et al. (2016); Demkina et al. (2023); Chopyk et al. (2020); Djurhuus (2017); Mueller et al. (2014); Stojan et al. (2023); Baron et al. (2015); Fecteau (2022); Hinthong et al. (2024); Anderson and Thompson (2022))	1	(Deshmukh et al. (2019))	0		0		1	(Lever et al. (2015))
Cellulose nitrate	4	(Walden et al. (2017); Djurhuus (2017); Brandt and Albertsen (2018); Sthapit et al. (2022))	4	(Gensberger et al. (2014); Amirhooshang et al. (2014); Uprety et al. (2024); Zhang et al. (2019))	1	(Oueslati. (2022))	0		0	
Cellulose acetate	0		1	(Kacprzak et al. (2015))	0		0		0	
Polycarbonate	6	(Djurhuus (2017); Cavallaro et al. (2024); Vierheilig et al. (2015); Fisher et al. (2020); Yun et al. (2016); Abreu‐Silva et al. (2023))	4	(Cao et al. (2018); Brooks et al. (2015); Fernández‐Baca. (2021); Consonni et al. (2021))	0		0		0	
Porous glass	0		0		1	(Djurhuus (2017))	2	(Uprety et al. 2023, 2024)	0	
Glass fiber	0		0		1	(Watanabe et al. (2020))	0		0	
Nylon	2	(Echeverry‐Gallego et al. (2024); Yang et al. (2020))	0		0		0		0	
Vinyl	1	(Djurhuus (2017))	0		0		0		0	
Alumina	1	(Mueller et al. (2014))	0		0		0		0	
Syringe	2	(Lee et al. (2019); Kurniawinata et al. (2022))	0		1	(Watanabe et al. (2020))	0		0	

The most used membrane type was PES (used in 14 studies out of 53, 26.41%), followed by the polycarbonate type (*n* = 10, 18.86%). Regardless of the membrane type, small pores of 0.2–0.6 µm were preferred all around included literature. The 0.22 µm membranes were mostly used (*n* = 23; 43.39%), followed by the 0.45 µm membranes (*n* = 13, 24.52%). Larger‐sized pores were used by (Lever et al. 2015) for larger, algae cells filtration. Filtration using a syringe was preferred only by three of the researchers, two of them using smaller pores: 0.2 µm (Kurniawinata et al. 2022) and 0.22 µm (Lee et al. 2019), while the other one applied a larger filtration unit, 0.7 µm (Watanabe et al. 2020).

### 3.3 Pre‐Extraction

Samples were prepared for extraction either with the help of an enzymatic pretreatment (*n* = 17; 32.07%), physical (*n* = 44; 83.01%), or detergent pretreatment (*n* = 6; 11.32%).

The enzymatic pretreatment involved cell lysis using protease (Walden et al. 2017), proteinase K (Lee et al. 2019; Demkina et al. 2023; Chopyk et al. 2020; Djurhuus. 2017; Gensberger et al. 2014; Shi et al. 2020; Schurig et al. 2024; Cavallaro et al. 2024; Mueller et al. 2014), lysozyme (Lee et al. 2019; Demkina et al. 2023; Chopyk et al. 2020; Djurhuus. 2017; Gensberger et al. 2014; Shi et al. 2020; Cavallaro et al. 2024; Mueller et al. 2014; Putri et al. 2021), or lysostaphin (Chopyk et al. 2020) added to the lysis buffer included in most extraction kits, for a supplemental cell lysis and better genetic material recovery. A variable number of different enzymes were used (1–4), but authors used one enzyme (*n* = 9; 16.98%), two enzymes (*n* = 4; 7.54%), three enzymes (*n* = 1; 1.88%), and four enzymes (*n* = 1; 1.88%).

Physical pretreatment involved either bead‐beating, centrifugation, vortexing, sonication, heating, freeze‐thaw cycles, documented in Table 3. The most common physical pretreatment was beads beating (*n* = 19; 35.84%), followed closely by centrifugation (*n* = 17; 32.07%) and processing of the sediment.

**Table 3:** Pre‐extraction methods: enzymatic pretreatments and buffers used for filter membranes; extraction methods; post‐extraction purposes of the studies.

Pre‐extraction									Extraction			Post‐extraction		
Buffer used	No. of studies	Reference	Enzymatic pretreatment (number of enzymes used)	No. of studies	Reference	Pretreatment	No. of studies	Reference	Kit	No. of studies	Reference	Purpose	No. of studies	Reference
Kit lysisbuffer	8	(Walden et al. (2017); Watanabe et al. (2020); Yasar et al. (2021); Cao et al. (2018); Anderson and Thompson (2022); Fernández‐Baca (2021); Marti et al. (2014); Zavarzina et al. (2022))	1	9	(Walden et al. (2017); Demkina et al. (2023); Djurhuus (2017); Schurig et al. (2024); Mueller et al. (2014); Stojan et al. (2023); Yasar et al. (2021); Oueslati. (2022); Fernández‐Baca. (2021))	beads	19	(Lee et al. (2019); Wijaya. (2023); Bautista‐de los Santos et al. (2016); Walden et al. (2017); Lever et al. (2015); Djurhuus. (2017); Shi et al. (2020); Schurig et al. (2024); Cavallaro et al. (2024); Vierheilig et al. (2015); Stojan et al. (2023); Cao et al. (2018); Baron et al. (2015); Hinthong et al. (2024); Anderson and Thompson (2022); Brandt and Albertsen (2018); Fernández‐Baca. (2021); Bae et al. (2019); Echeverry‐Gallego et al. (2024))	Commercial	40	(Wijaya. (2023); Walden et al. (2017); Demkina et al. (2023); Chopyk et al. (2020); Gensberger et al. (2014); Shi et al. (2020); Schurig et al. (2024); Cavallaro et al. (2024); Mueller et al. (2014); Putri et al. (2021); Stojan et al. (2023); Yasar et al. (2021); Cao et al. (2018); Uprety et al. (2023); Arsand et al. (2020); Fisher et al. (2020); Brooks et al. (2015); Bian et al. (2023); Huang et al. (2017); Fecteau. (2022); Hinthong et al. (2024); Anderson and Thompson (2022); Brandt and Albertsen (2018); Sthapit et al. (2022); Amirhooshang et al. (2014); Fernández‐Baca (2021); Bae et al. (2019); Smalls et al. (2023); Marti et al. (2014); Mochware et al. (2024); Yao et al. (2024); Uprety et al. (2024); Echeverry‐Gallego et al. (2024); Yun et al. (2016); Zavarzina et al. (2022); Yang et al. (2020); Abreu‐Silva et al. (2023); Wirajana et al. (2024); Kacprzak et al. (2015); Consonni et al. (2021))	16S	33	(Wijaya (2023); Bautista‐de los Santos et al. (2016); Walden et al. (2017); Watanabe et al. (2020); Kurniawinata et al. (2022); Demkina et al. (2023); Djurhuus (2017); Gensberger et al. (2014); Vierheilig et al. (2015); Kambura et al. (2016); Stojan et al. (2023); Yasar et al. (2021); Uprety et al. (2023); Deshmukh et al. (2019); Oueslati (2022); Baron et al. (2015); Fisher et al. (2020); Bian et al. (2023); Huang et al. (2017); Fecteau (2022); Hinthong et al. (2024); Anderson and Thompson (2022); Brandt and Albertsen (2018); Amirhooshang et al. (2014); Bae et al. (2019); Yao et al. (2024); Uprety et al. (2024); Echeverry‐Gallego et al. (2024); Yun et al. (2016); Zavarzina et al. (2022); Yang et al. (2020); Abreu‐Silva et al. (2023); Wirajana et al. (2024))
PBS	8	(Wijaya (2023); Chopyk et al. (2020); Deepnarain et al. (2020); Deshmukh et al. (2019); Brooks et al. (2015); Huang et al. (2017); Smalls et al. (2023); Mochware et al. (2024))	2	4	(Kurniawinata et al. (2022); Gensberger et al. (2014); Shi et al. (2020); Cavallaro et al. (2024))	centrifugation	17	(Bautista‐de los Santos et al. (2016); Djurhuus. (2017); Mueller et al. (2014); Kambura et al. (2016); Stojan et al. (2023); Deepnarain et al. (2020); Cao et al. (2018); Oueslati (2022); Hoorzook and Barnard (2022); Fisher et al. (2020); Bian et al. (2023); Huang et al. (2017); Hinthong et al. (2024); Anderson and Thompson (2022); Amirhooshang et al. (2014); Fernández‐Baca. (2021); Mochware et al. (2024))	In‐house	15	(Lee et al. (2019); Bautista‐de los Santos et al. (2016); Watanabe et al. (2020); Lever et al. (2015); Kurniawinata et al. (2022); Djurhuus. (2017); Shi et al. (2020); Vierheilig et al. (2015); Kambura et al. (2016); Stojan et al. (2023); Deepnarain et al. (2020); Deshmukh et al. (2019); Oueslati (2022); Hoorzook and Barnard (2022); Baron et al. (2015))	qPCR	23	(Lee et al. (2019); Lever et al. (2015); Demkina et al. (2023); Gensberger et al. (2014); Shi et al. (2020); Schurig et al. (2024); Deepnarain et al. (2020); Yasar et al. (2021); Uprety et al. (2023); Arsand et al. (2020); Deshmukh et al. (2019); Brooks et al. (2015); Fecteau (2022); Sthapit et al. (2022); Fernández‐Baca (2021); Bae et al. (2019); Smalls et al. (2023); Marti et al. (2014); Mochware et al. (2024); Uprety et al. (2024); Abreu‐Silva et al. (2023); Kacprzak et al. (2015); Consonni et al. (2021))
Tris EDTA	2	(Kambura et al. (2016); Oueslati (2022))	3	1	(Lee et al. (2019))	CTAB	1	(Vierheilig et al. (2015))				Plasmids	1	(Yasar et al. (2021))
			4	1	(Chopyk et al. (2020))	SDS	4	(Chopyk et al. (2020); Shi et al. (2020); Deshmukh et al. (2019); Oueslati. (2022))				ddPCR	1	(Cao et al. (2018))
			not mentioned	2	(Shi et al. (2020); Yang et al. (2020))	TritonX	1	(Demkina et al. (2023))				DNA quantification	1	(Putri et al. (2021))
						heat	4	(Lever et al. (2015); Schurig et al. (2024); Sthapit et al. (2022); Abreu‐Silva et al. (2023))				method comparison	1	(Mueller et al. (2014))
						vortex	2	(Oueslati. (2022); Mochware et al. (2024))				shotgun sequencing, metagenomic assembly	1	(Chopyk et al. (2020))
						sonication	1	(Shi et al. (2020))						
						Freeze‐thaw	1	(Lever et al. (2015))						
						not mentioned	1	(Yang et al. (2020))						

The usage of different tensioactive agents such as CTAB (Cetyltrimethylammonium bromide), or detergents like SDS (sodium dodecyl sulfate), or nonionic surfactants such as Triton X, were documented in Table 3.

In some cases, the filter membranes were placed into a buffer before the extraction. The most commonly used buffers were PBS—phosphate‐buffered saline (*n* = 8; 15.09%), and the lysis buffers provided with the commercial extraction kits (*n* = 8; 15.09%).

### 3.4 Extraction

Most of the authors extracted bacterial DNA with commercial kits (*n* = 40; 75.47%) and others by using in‐house methods (15, 28.30%). The in‐house methods are different adaptations of CTAB—cetyltrimethylammonium bromide/SDS—sodium dodecyl sulfate method (Kurniawinata et al. 2022; Vierheilig et al. 2015), or varieties of the phenol/chloroform method. All in‐house methods were used either to perform qPCR and/or to extract 16S ribosomal RNA. DNA obtained with kits showed higher amplification success, especially in low biomass samples (Bautista‐de los Santos et al. 2016; Watanabe et al. 2020; Vierheilig et al. 2015; Kambura et al. 2016; Stojan et al. 2023; Deepnarain et al. 2020).

For plasmid extraction, the preferred extraction method was by using the MO BIO by Qiagen, PowerWater Sterivex DNA Isolation Kit (Yasar et al. 2021). For 16S ribosomal RNA, most of the authors used commercial kits (*n* = 23, 43.39%), and in‐house methods (*n* = 9; 16.98%). The extraction for qPCR was conducted using in‐house adapted methods (*n* = 4; 7.54%), and by using commercial kits (*n* = 19; 35.84%). Other reasons for extraction were shotgun sequencing (Chopyk et al. 2020), ddPCR (Cao et al. 2018), and comparison of the DNA yield obtained by several methods (Mueller et al. 2014) (Table 4).

**Table 4:** Commercial kits used for bacterial DNA extraction.

Commercial kit name	No. of studies	Reference
Qiagen, DNeasy PowerWater Kit	8	(Putri et al. (2021); Stojan et al. (2023); Uprety et al. (2023); Hinthong et al. (2024); Brandt and Albertsen (2018); Smalls et al. (2023); Uprety et al. (2024); Abreu‐Silva et al. (2023))
MP Biomedicals, FastDNA SPIN Kit for Soil	7	(Shi et al. (2020); Uprety et al. (2023); Fecteau (2022); Brandt and Albertsen (2018); Uprety et al. (2024); Zavarzina et al. (2022); Yang et al. (2020))
MO BIO by Qiagen, PowerWater Sterivex DNA Isolation Kit	5	(Walden et al. (2017); Djurhuus. (2017); Yasar et al. (2021); Yun et al. (2016); Kacprzak et al. (2015))
MO BIO by Qiagen, PowerSoil DNA Isolation Kit	3	(Walden et al. (2017); Yasar et al. (2021); Arsand et al. (2020))
Qiagen, DNeasy Blood & Tissue Kit	3	(Wijaya (2023); Marti et al. (2014); Echeverry‐Gallego et al. (2024))
Qiagen, DNeasy PowerSoil Pro Kit	3	(Wijaya (2023); Demkina et al. (2023); Echeverry‐Gallego et al. (2024))
Qiagen, QIAamp Fast DNA Mini Kit	3	(Walden et al. (2017); Brooks et al. (2015); Amirhooshang et al. (2014))
Qiagen, QIAamp Fast DNA Stool Mini Kit	3	(Walden et al. (2017); Demkina et al. (2023); Brooks et al. (2015))
Phigenics, Ultra Rapid DNA Extraction (P.U.R.E.)	1	(Fisher et al. (2020))
Biomeme Field Sample Prep Kit (M1)	1	(Fernández‐Baca. (2021))
Epicenter, WaterMaster DNA Purification Kit	1	(Gensberger et al. (2014))
GeneRite, DNA‐EZ Extraction kit	1	(Cao et al. (2018))
Kanto Chemical Co., Cica Geneus DNA extraction kit	1	(Sthapit et al. (2022))
LSBio, Soil Genomic DNA Isolation Kit	1	(Demkina et al. (2023))
Lucigen/Epicentre, MasterPure Complete DNA and RNA Purification Kit	1	(Mueller et al. (2014))
MagPurix 12 s Automated Nucleic Acid Purification System	1	(Huang et al. (2017))
MagPurix Bacterial DNA Extraction Kit	1	(Huang et al. (2017))
Minerva Biolabs, AquaScreen FastExtract	1	(Consonni et al. (2021))
MO BIO by Qiagen, UltraClean Soil DNA Isolation Kit	1	(Bae et al. (2019))
New England Biolabs, Monarch HMW DNA Extraction Kit for Tissue	1	(Demkina et al. (2023))
Omega Bio‐Tek, E.Z.N.A. SoilDNA Kit	1	(Bian et al. (2023))
Omega Bio‐Tek, E.Z.N.A. Stool DNA Kit	1	(Yao et al. (2024))
Qiagen, QIAamp DNA Microbiome Kit	1	(Demkina et al. (2023))
Qiagen, QIAamp DSP DNA Mini Kit	1	(Chopyk et al. (2020))
Qiagen, QIAamp PowerFecal DNA Kit	1	(Demkina et al. (2023))
Sisco Research Laboratories, BioLit Genomic DNA Extraction Mini Kit	1	(Wirajana et al. (2024))
TaKaRa, MiniBEST Bacteria Genomic DNA Extraction Kit Ver.3.0	1	(Shi et al. (2020))
TaKaRa, NucleoMag DNA/RNA Water kit	1	(Anderson and Thompson (2022))
Thermo Fisher Scientific, PureLink Microbiome DNA Purification Kit	1	(Demkina et al. (2023))
TIANGEN, Magnetic Genomic DNA Kit	1	(Shi et al. (2020))
Xpedite Diagnostics, SwiftX DNA	1	(Schurig et al. (2024))
Xpedite Diagnostics, SwiftX Toolbox	1	(Schurig et al. (2024))
Xpedite Diagnostics, SwiftX ParaBact	1	(Schurig et al. (2024))
Xpedite Diagnostics, SwiftX Swabs	1	(Schurig et al. (2024))
Zymo Research, Quick‐DNA Fecal/Soil Microbe Microprep	1	(Mochware et al. (2024))
Zymo Research, ZymoBIOMICS DNA Kit	1	(Anderson and Thompson (2022))

The commercial extraction kits preferred by the authors are various, the most common ones being Qiagen, Dneasy PowerWater Kit (*n* = 8, 15.09%), MP Biomedicals, FastDNA SPIN Kit for Soil (*n* = 7, 13.20%), and MO BIO by Qiagen, PowerWater Sterivex DNA Isolation Kit (*n* = 5; 9.43%).

## 4 Discussion

Our study shows the multiple variations in the protocols used by different researchers when analyzing water samples. These variations might occur because of the absence of a general consensus regarding the optimal methods used for sampling and filtration of water samples, followed by extraction to recover bacterial genetic material.

**Figure 2:**
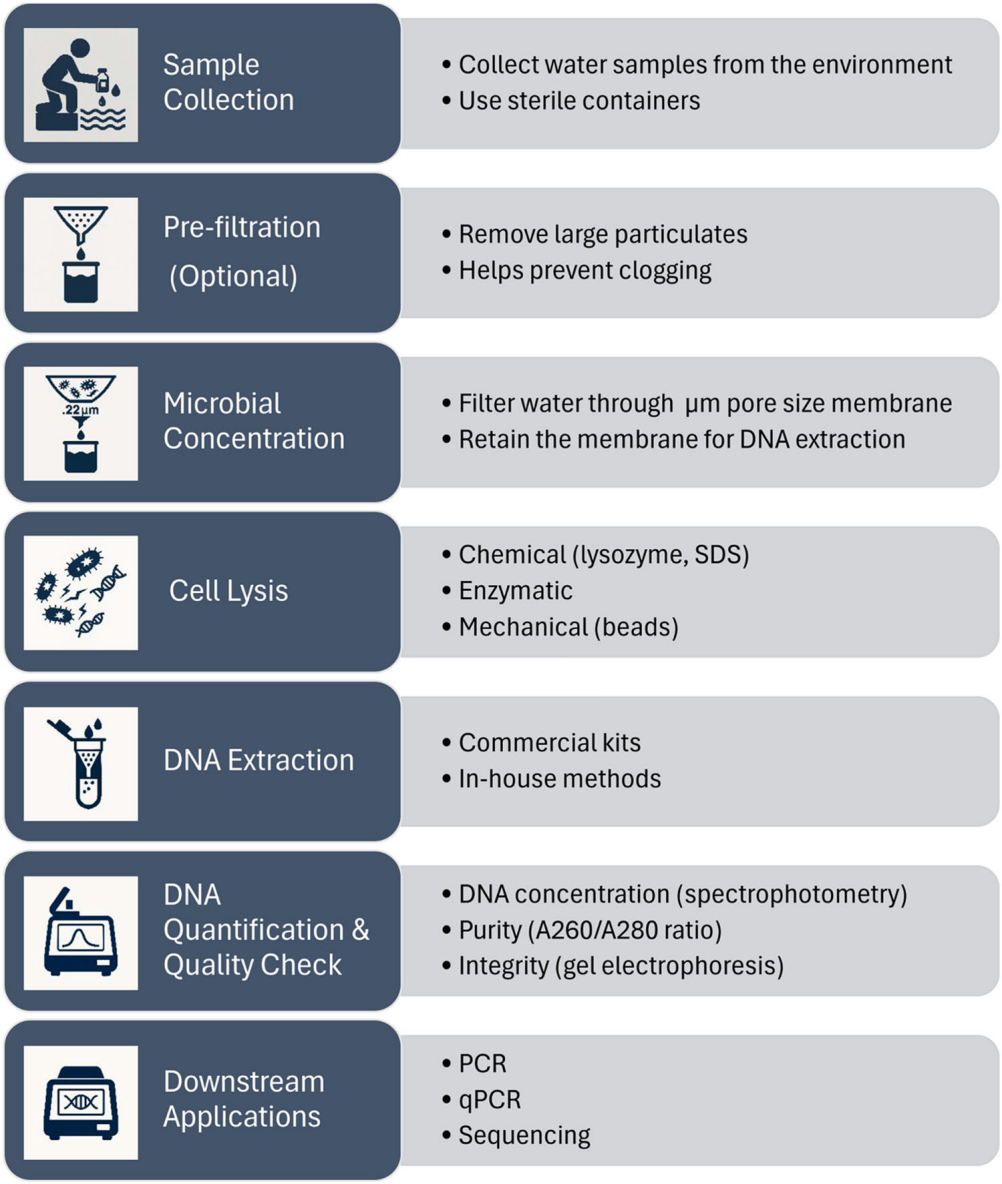
The recommended workflow of water filtration.

Figure 2 shows the recommended workflow of membrane filtration for water samples.

### 4.1 Sampling

Water sampling for bacterial DNA extraction is an important step in the correct assessment of the microbial communities present in various bodies of water, as suggested by Madrid and Zayas (Madrid and Zayas 2007). The pre‐analytic step involves the actual sampling, which is usually done outside the laboratory, but also the transportation and the storage of the sample in appropriate conditions (Madrid and Zayas 2007). Most of the authors from the included studies preferred using recipients of different volumes, according to the quantity needed for research, which might be also related to better logistics and time management, as compared to horizontal water samplers or pumps, which would need a power source to function. The location of the sample, the volume, or the number of replicates could be important for optimal recovery of genetic material, as referred by Staley et al. 2015 (Staley et al. 2015). The Wildco, Model 1960‐H65 (Yulee, FL) horizontal water sampler (Walden et al. 2017), brings the advantage of allowing sampling from specific depths and choosing the desired stratification level of deep waters, with a total volume of 6.2 (Beta Plus Bottle Only— 2025). (Table 5).

**Table 5:** Comparison between water collection methods.

Criteria	Small containers	Pumps
Sample volume	Limited (100 mL – 1 L)	Can collect large volumes (up to 100 + L) (Planquette and Sherrell 2012)
Effort required	Manual sampling	Easier for collecting large volumes with less effort (Wilde et al. 1999)
Field portability	Very portable, no power required (Wilde et al. 1999)	Requires equipment (manual or powered) (Wilde et al. 1999)
Sampling depth/access	Surface only or shallow sampling (Madrid and Zayas 2007)	Enables sampling from depth (e.g., wells, reservoirs) (Wilde et al. 1999)
Contamination risk	Higher if bottle is opened/handled incorrectly (Wilde et al. 1999)	Lower if using closed systems or sterile tubing (Wilde et al. 1999)
Speed of collection	Slower for large volumes (multiple containers needed)	Faster for large‐volume collection (Planquette and Sherrell 2012)
Suitability for remote sites	High (Wilde et al. 1999)	Varies (manual pumps good; powered ones need power source) (Wilde et al. 1999)
Sterility control	Must sterilize each container	Requires sterilized pump/tubing (more complex cleaning)
Cost	Low	Moderate (depends on pump type and durability)
Ideal use case	Quick samples, low volumes (Wilde et al. 1999)	Large volume collection, hard‐to‐reach water sources (Wilde et al. 1999)

### 4.2 Filtration

The filtration of the samples was usually done using membrane filters with smaller pores, to concentrate bacterial cells. Larger pores were used due to high turbidity of the sample (Uprety et al. 2023), to remove particulate matter (Arsand et al. 2020), or to remove algal cells (Lever et al. 2015). The preferred membrane was the 0.22 μm pore‐size, even though it was shown that it does not offer any supplemental advantages from the 0.45 μm membrane (Carter 1996). If liquids with different viscosities are filtered, membrane parameters such as flux or resistance should be considered, as demonstrated by Dizge et al. 2011, (Dizge et al. 2011). The volume of filtered water is another parameter that should be considered, depending on the concentration of bacteria present. Lower volumes are recommended if the water is highly concentrated in larger particles, which might clog the membrane filters, or if a vacuum setup is used, while larger volumes are more suitable for on‐site filtration (Hozalski et al. 2024). Across the included studies, comparisons between membrane pore sizes were limited but informative. Although the 0.22 μm filters were the most commonly used (*n* = 23; 53.48%), a few studies directly compared their performance with 0.45 μm filters, reporting minimal differences in bacterial DNA recovery when sample turbidity was low. In contrast, larger pore sizes were preferred in high‐particulate or algal‐rich waters to prevent clogging, albeit with a potential loss in bacterial capture efficiency (Table 6).

**Table 6:** Comparison of membrane filtration methods for further bacterial DNA extraction.

Criteria	0.2 µm/0.22 µm	0.45 µm	Larger pore size
Quantity filtered	Lower, depending on the foreign particle concentration (Hozalski et al. 2024)	Lower, depending on the foreign particle concentration (Hozalski et al. 2024)	Higher (Takasaki et al. 2021)
Microorganisms	Retains most bacteria, fungi, but also larger cells (Goswami and Pugazhenthi 2020)	Retains most bacteria, fungi, but also larger cells (Goswami and Pugazhenthi 2020)	Retains only larger cells (fungi, algae) (Lever et al. 2015; Takasaki et al. 2021)
Advantages	Allows filtration for clean water Only one filtration necessary DNA can be extracted directly from the filter The filters can be cultured (for CFU quantification) (Hozalski et al. 2024)	Allows filtration for clean water Only one filtration necessary DNA can be extracted directly from the filter The filters can be cultured (for CFU quantification) (Hozalski et al. 2024)	Allows filtration for impure water Pre‐filtration might improve DNA yield (Takasaki et al. 2021)
Disadvantages	Might clog easily Lower volumes if a vacuum system is used (Hozalski et al. 2024)	Might clog easily Lower volumes if a vacuum system is used (Hozalski et al. 2024) Some bacteria may not be captured (Wang et al. 2007)	Requires filtration with smaller pores membranes for bacterial retention (Wang et al. 2007)
Infrastructure	Requires vacuum systems (Criscuoli et al. 2013)	Requires vacuum systems (Criscuoli et al. 2013)	Requires vacuum systems Requires another filtration process for bacterial retention
Ideal use case	Suitable for low quantities of water (up to 1 L), retains bacteria (Hozalski et al. 2024)	Suitable for low quantities of water (up to 1 L), retains bacteria (Hozalski et al. 2024)	Suitable for pre‐filtration (Takasaki et al. 2021)

### 4.3 Pre‐Extraction

Some authors were preparing the samples before extraction by using either an enzymatic or a physical pretreatment.

Proteases are enzymes used to help the degradation of proteins and nucleases that might damage DNA. According to Eychner et al. (2015), there are multiple commercially available protases, such as papain and bromelain, two cysteine proteases that have hydrolyze DNases, being able to cleave amino acids, resulting in protein degradation. Proteinase K is a type of a protease that we decided to treat separately due to its popularity among authors. Proteinase K is a usually stable enzyme extracted from a fungus, and it is used together with a lysis buffer (usually provided by the commercial extraction kit), with effects on proteins of the cell walls, including the degradation of the peptidoglycan, resulting in better DNA recovery (Eychner et al. 2015; Gautam 2022).

Lysozyme is also a widely used enzyme due to its hydrolytic effect, especially in Gram‐positive bacteria. Lysozyme is able to break the bonds between NAM and NAG, altering the cellular wall of bacteria, increasing the DNA yield obtained after extraction, as Zhang et al. demonstrated in their research (Zhang et al. 2023).

One author used lysostaphin (Chopyk et al. 2020). The effect of lysostaphin is on the pentaglycine bridges of the peptidoglycan (Galeano et al. 2025), yet better results, according to Zhao et al. 2012, are in combination with lysozyme (Zhao et al. 2012) (Table 7).

**Table 7:** Frequently used enzymes to enhance DNA yield.

Enzyme	Targets	Advantages	Disadvantages
Proteinase K	Proteins (not cell walls) (Shahriar et al. 1970)	Removes nucleases, enhances DNA purity (Shahriar et al. 1970)	Doesn't lyse cells; needs other lysis steps (Shahriar et al. 1970)
Lysozyme	Gram‐positive bacteria (Zhang et al. 2023)	Effective lysis for thick‐peptidoglycan bacteria (Zhang et al. 2023)	Poor on Gram‐negative; incomplete in mixed samples (Zhang et al. 2023)
Lysostaphin	Staphylococcus spp (Galeano et al. 2025).	Highly effective against Staphylococcus (Galeano et al. 2025)	Very narrow spectrum (Galeano et al. 2025), expensive

The most common physical pretreatment used in the included studies is bead‐beating. The mechanical effect of beads should be limited to cell disruption, not the DNA itself, therefore choosing the correct intensity, speed and bead material could be crucial in obtaining the best DNA yield possible. Beads of different materials are available in the market (ceramic, glass). To protect the DNA, the bead‐beating process should be supplemented with a lysis buffer and an enzymatic treatment (such as proteinase K), as demonstrated by some researchers (Yuan et al. 2015; Bürgmann et al. 2001). Excessive bead beating can fragment genomic DNA, which is particularly problematic when preserving large, intact molecules for sequencing. Plasmids also can be damaged by intensive physical methods. Thus, diagnostic targets may be lost, impacting environmental surveillance. Also, the microbial diversity findings can be impaired, affecting ecological assessments (Smalla et al. 2015).

Centrifugation is a method used by many of the authors, an advantage being the affordability of the method, the centrifuge being an indispensable piece in every laboratory infrastructure. Centrifugation can be used to separate heavy particles that might be present in water, such as dirt, sand or other debris. Robe et al. suggests a low‐speed centrifugation varying from 500 x g to 1000 x g to separate these large particles into a pellet, followed by a more intense centrifugation, at 10,000 x g to accomplish bacterial fraction. However, some bacterial cells might be lost during these steps, due to their variation in buoyancy or due to their arrangement in clusters or chains (Robe et al. 2003).

Samples that undergo any chemical pretreatment need to be vortexed to remove clumps and to homogenize the suspension, for better results (Zoetendal et al. 2001; Smith et al. 2011). Another study relates that vortexing can be used in combination with different heat and time settings (1400 rpm at 75°C, for up to 90 min), with 2% SDS. It was shown that by using the combination, the DNA recovery can be increased significantly (Pasha et al. 2020).

To increase cell lysis, sonication combined with physical (bead‐beating) or chemical (proteinase K, commercial lysis buffers provided with extraction kits) methods can be used (Rantakokko‐Jalava and Jalava 2002). Some studies found that sonication is efficient only with high molecular weight DNA and the outcome is influenced by the type of instrument used (de Lipthay et al. 2004; Westergaard et al. 2001).

Freeze‐thaw cycles can improve the DNA yield on some samples, according to deLipthay JR et al (de Lipthay et al. 2004). Other research revealed that multiple freeze‐thaw cycles can damage large DNA in vitro (Shao et al. 2012), while in nature, repeated cycles are demonstrated to influence some functions of the bacterial cells (e.g. the gene expression of some genes, affecting the denitrifying potential of some bacteria) (Sharma et al. 2006).

Cetyltrimethylammonium bromide is a cationic surfactant and is described by Salton et al (Salton 1951). as a detergent with high affinity for bacterial cells, having a bactericidal effect, being able to degrade proteins and to release various cellular constituents in similar amounts as boiling does (Salton 1951; Nakata et al. 2011).

Sodium dodecyl sulfate (SDS) in different concentrations (0.5%, 1%, 2%) can be used as a lysis buffer, or in addition to another lysis buffer, or together with other enzymes (proteinase K, lysozyme) (Goldenberger et al. 1995; Zaporozhenko et al. 2006). SDS is an amphiphilic molecule that forms micelles in aqueous solutions and binds to polypeptides, inducing a negative charge that disturbs the polarity of the cell membrane (Costas 1995; Hammouda 2013).

Non‐ionic surfactants belonging to Triton X series were used by one of the cited authors (Demkina et al. 2023), as a lysis buffer, together with other agents (TrisHCl, EDTA and NaCl). Some authors suggest a higher efficiency in recovering various fractions of DNA by using Triton X (Ban et al. 2013).

Regarding DNA extraction, only a subset of studies quantified yield and purity. Reported total DNA yields ranged from 1 ng/μL to over 100 ng/μL depending on the extraction method and sample type. Commercial kits tended to produce more consistent A260/280 ratios (typically ~1.8–2.0), indicating higher DNA purity, whereas in‐house protocols showed greater variability (Table 8).

**Table 8:** Detergents used to improve cell lysis and DNA extraction.

Detergent	Type	Advantages	Limitations
SDS	Anionic	Strong lysis, denatures proteins including nucleases, compatible with proteinase K (JF and Russell 2001)	Aggressively denature proteins, can inhibit downstream enzymatic reactions if not cleaned, weak on Gram‐positive bacteria (Ausubel 2009)
Triton X‐100	Non‐ionic	Selective lysis of eukaryotic cells or Gram‐negative bacteria, membrane solubilization, preserves DNA integrity (Helenius and Simons 1975)	Weak on Gram‐positive bacteria or tough cells (Wilson 2001)
CTAB	Cationic	Good for processing polysaccharide‐rich environmental samples, the polysaccharide removal leading to good DNA purity (Porebski et al. 1997)	Requires high‐salt buffers, multiple steps, toxicity, can co‐precipitate impurities (Wilson 1997)

### 4.4 Extraction

Among all 53 studies included in this review, 36 different commercial extraction kits were used, which shows a great variety in the market regarding options for DNA extraction, although only four of them have the word “water” in the kit's name. This shows the huge versatility of the commercial extraction kits when it comes to extracting genetic material from bacteria found in nature, including water. Further research is needed to assess the cost‐efficiency of each type of extraction mentioned.

In‐house extraction methods are also an option, according to some of the studies cited. The methods described are adaptations of CTAB/SDS method, or varieties of the phenol/chloroform method, or other adapted protocols, each described by the authors who used them (Lee et al. 2019; Bautista‐de los Santos et al. 2016; Watanabe et al. 2020; Lever et al. 2015; Kurniawinata et al. 2022; Djurhuus. 2017; Shi et al. 2020; Vierheilig et al. 2015; Kambura et al. 2016; Stojan et al. 2023; Deepnarain et al. 2020; Deshmukh et al. 2019; Oueslati. 2022; Hoorzook and Barnard 2022; Baron et al. 2015).

Both commercial kits and in‐house methods can be used for bacterial DNA extraction, each with distinct advantages and limitations. Commercial kits offer standardized protocols, ease of use, and compatibility with downstream applications such as qPCR and 16S rRNA analysis, though they can be costly and less flexible. In contrast, in‐house methods are more cost‐effective and adaptable, especially for hard‐to‐lyse bacteria, but are time‐consuming, require optimization, hazardous reagents, and may yield less reproducible results. The choice largely depends on sample type, resources, and intended downstream applications.

To note, the DNA extraction kit might be affected by the composition and diversity of the microbial community, and some kits might exclude certain bacteria with a robust cell wall that cannot be lysed through traditional methods (Galla et al. 2024; Ketchum 2018).

### 4.5 Suggested Good Practices in Writing an Article

For improving the transparency, we suggest the following good practices for writing the methodology section of an article on the subject of genetic material extraction from bacteria present in water:

- Mention the place of sampling, including the geographical coordinates, as well as the depth of collection;
- Include information about the type of water sampled (fresh water, salt water, and so on);
- Mention the quantity of each sample, the number of samples collected and the time frame of the sampling process;
- Briefly present the type of container used for the samples;
- If preservation methods are used, they should be noted;
- If filter membranes are used, add information about the type of filter (nitrocellulose, PES, and so on), pore size, the commercial name of it, the type of vacuum system used, as well as the quantity of the aliquot that actually ran through the filter.
- If the filters were placed on culture media, or enriched in any way, it is best to be disclosed;
- Any physical or chemical method used to detach bacteria from filter membranes are important;
- The pre‐extraction processing of the samples (bead‐beating, centrifugation, enzymatic pretreatment, and so on) is worth mentioning;
- Disclose the extraction method, including the commercial name of the extraction kit, and all the changes made to the original protocol. If in‐house methods are used, please describe them;
- The purpose of the extraction is important for future research and it should be presented;
- Any measurements of the DNA yield that were done, including the method and the numerical values should be added;
- Finally, a section of advantages and disadvantages of the methods used can be helpful for other researchers.

### 4.6 Limitations of the Review Process

Although data extraction followed a pre‐established protocol, there is missing data (non‐reported data) dispersed over the database. The missing data reflect the need for standardizing reporting in experimental studies.

Certainty of evidence was not assessed because none of the existing scales fit the particularities of our study.

The reporting of experimental data in the reviewed studies lack standardization, largely due to methodological heterogeneity. This variability can be justified by the wide range of research objectives, water sources, sample types, and analytical techniques employed across studies, which naturally require different approaches. Though, flexibility in methods can be advantageous, allowing adaptation to field conditions or novel challenges, reflecting the evolving nature of environmental molecular microbiology.

### 4.7 Implications of Results in Practice and Future Research

Data summarized in this systematic review might help researchers in choosing the appropriate methods for sampling water from different sources, finding membrane filtration methods, and discovering the culture‐free extraction methods that fit their purpose. As water filtration and DNA extraction are commonly used in studies that are researching the microbiological status of different bodies of water, the spectrum of possibilities is substantially wider, and it still remains a subject of further research.

Prospective developments may include the creation of robust, field‐adapted extraction platforms, improved integration with portable sequencing or amplification systems, and the development of universal protocols tailored to complex water matrices. Such progress will enhance the reliability, scalability, and accessibility of culture‐free microbial water analysis across both research and public health domains.

Future research should not only adopt standardized reporting practices but also include critical reflections, methodological justifications, and practical recommendations. Including such would enhance transparency, reproducibility, and comparability across the field, ultimately contributing to the consolidation of best practices in water microbiology research.

## 5 Conclusion

The review summarizes the current practices available for researchers wanting to study bacterial molecular characteristics from water samples. Frequently used practices were collecting water in sterile containers and in duplicates or triplicates, filtering smaller aliquots, usually below 1000 mL using the available membranes in the market, with larger pores to remove larger cells and debris, and smaller pores to capture and recover as much bacteria as possible. Preparing the sample for extraction usually requires some additional steps, either physical, chemical or both, to ensure a larger DNA recovery. The extraction can be done either with commercial kits or with in‐house methods, but every researcher should choose the right techniques based on the study's characteristics, access to resources and available logistics.

The heterogeneity of data among included studies highlights the need for standardizing reporting in experimental studies. Future studies should address missing aspects such as cost‐effectiveness analyses, direct comparisons between commercial kits and in‐house protocols, and field validation of standardized extraction procedures to improve reproducibility and practical applicability in diverse environmental settings.

## Author Contributions

**Radu Ovidiu Togănel:** writing – original draft, conceptualization, methodology, data curation, investigation, formal analysis. **Cristina Nicoleta Ciurea:** writing – review and editing, conceptualization, methodology, investigation, formal analysis. **Anca Cighir:** writing – review and editing, conceptualization, methodology, investigation, data curation, formal analysis. **Anca Delia Mare:** writing – review and editing. **Razvan Lucian Coșeriu:** writing – review and editing. **Camelia Vintilă:** writing – review and editing. **Dragoș Constantin Cucoranu:** writing – review and editing. **Adrian Man:** writing – review and editing, conceptualization, methodology, data curation, supervision, validation.

## Conflicts of Interest

The authors declare no conflicts of interest.

## Supporting information

suplementary material.

## Data Availability

Data sharing not applicable to this article as no datasets were generated or analyzed during the current study. Data will be made available on request.
