# Supplementary figures and images for: The Latest Practices in Culture‐Free Detection of Bacteria in Water, From Sampling to Membrane Filtration and DNA Extraction: A Systematic Review
Source: Microbiologyopen. 2025 Nov 4;14(6):e70119. doi: 10.1002/mbo3.70119 (PMC12586351; doi:10.1002/mbo3.70119)

1. Search:

- Web of Science


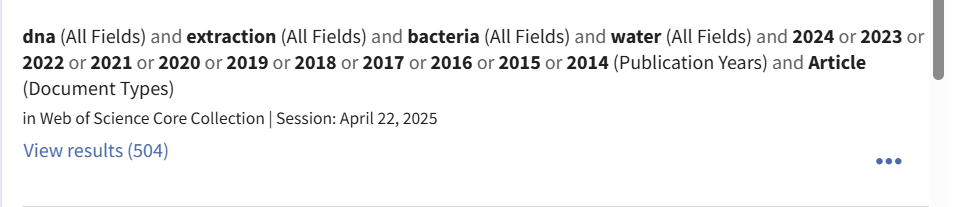


- PubMed


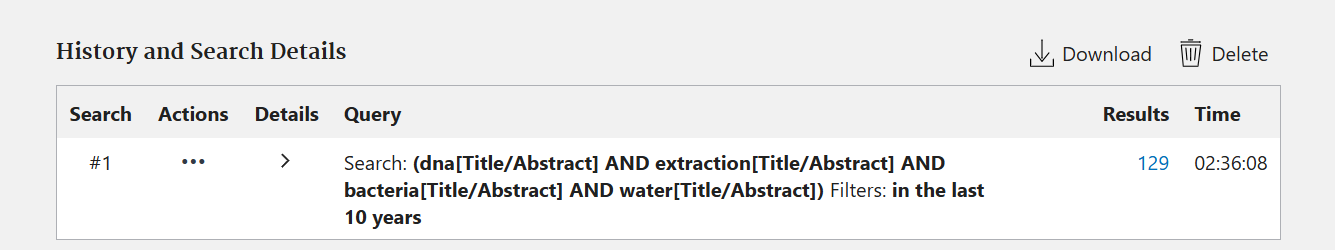


1. PRISMA 2020 Checklist


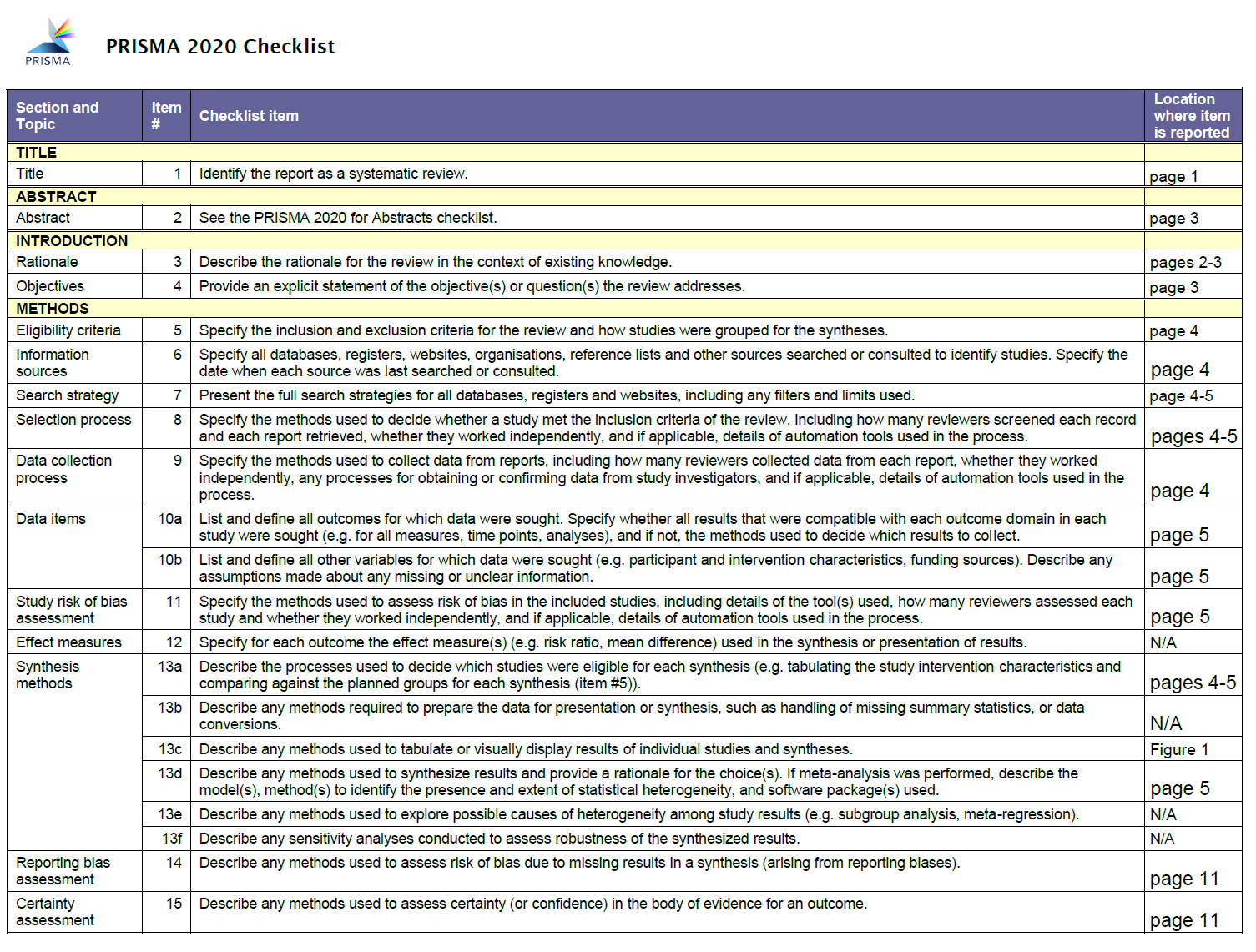


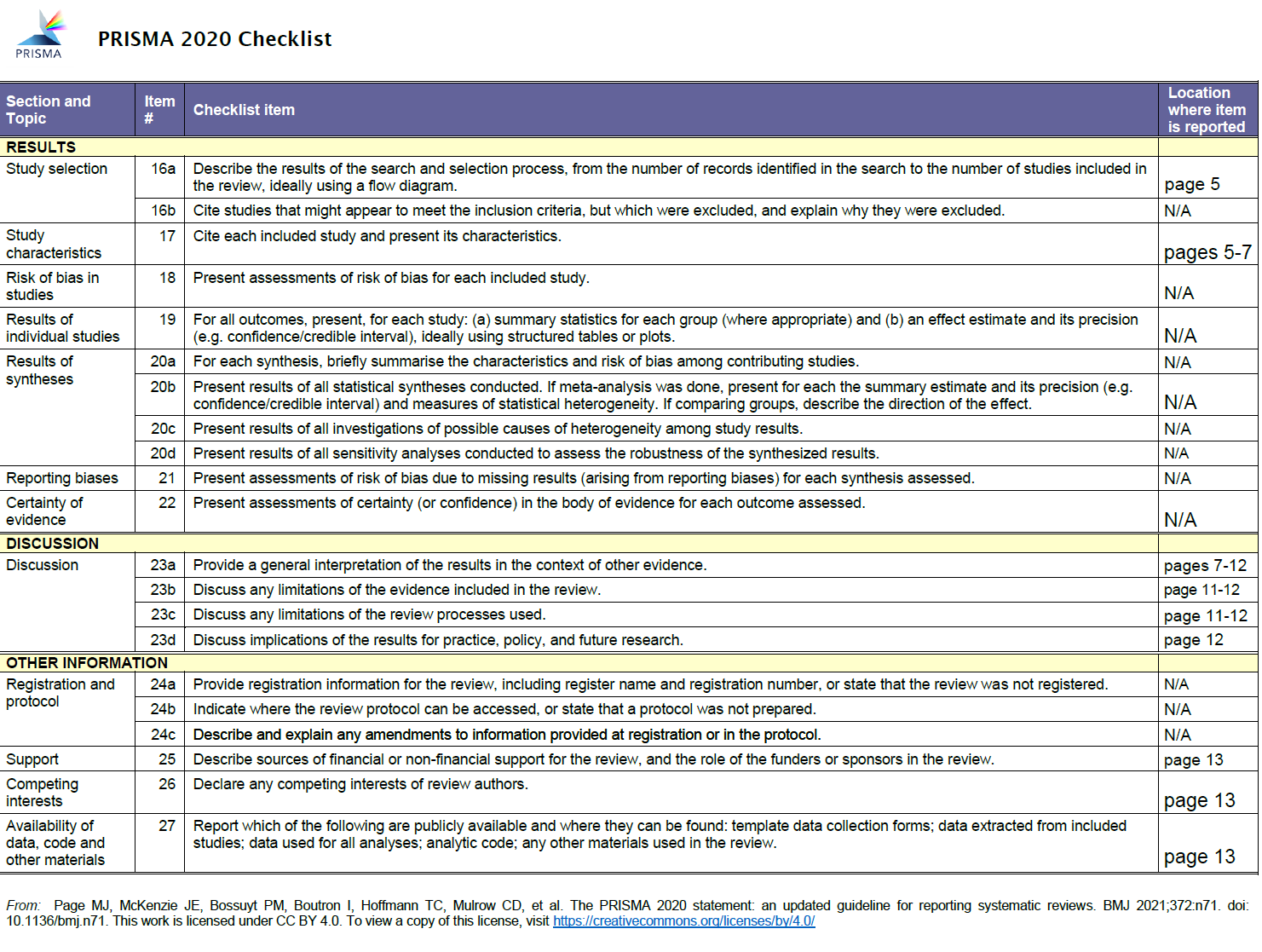

Supplement: Supplementary file 1 — suplementary material. [file MBO3-14-e70119-s001.docx]
